# Imaging DNA Damage Repair In Vivo After ^177^Lu-DOTATATE Therapy

**DOI:** 10.2967/jnumed.119.232934

**Published:** 2020-05

**Authors:** Edward O’Neill, Veerle Kersemans, P. Danny Allen, Samantha Y.A. Terry, Julia Baguña Torres, Michael Mosley, Sean Smart, Boon Quan Lee, Nadia Falzone, Katherine A. Vallis, Mark W. Konijnenberg, Marion de Jong, Julie Nonnekens, Bart Cornelissen

**Affiliations:** 1 CRUK/MRC Oxford Institute for Radiation Oncology, Department of Oncology, University of Oxford, Oxford, United Kingdom; 2 Department of Imaging Chemistry and Biology, King’s College London, London, United Kingdom; 3 Department of Radiology and Nuclear Medicine, Erasmus MC, Rotterdam, The Netherlands; 4 Department of Molecular Genetics, Erasmus MC, Rotterdam, The Netherlands; and; 5 Oncode Institute, Erasmus MC, Rotterdam, The Netherlands

**Keywords:** ^177^Lu-DOTATATE, γH2AX, SPECT, DNA damage, neuroendocrine cancer

## Abstract

Molecular radiotherapy using ^177^Lu-DOTATATE is a most effective treatment for somatostatin receptor–expressing neuroendocrine tumors. Despite its frequent and successful use in the clinic, little or no radiobiologic considerations are made at the time of treatment planning or delivery. On positive uptake on octreotide-based PET/SPECT imaging, treatment is usually administered as a standard dose and number of cycles without adjustment for peptide uptake, dosimetry, or radiobiologic and DNA damage effects in the tumor. Here, we visualized and quantified the extent of DNA damage response after ^177^Lu-DOTATATE therapy using SPECT imaging with ^111^In-anti-γH2AX-TAT. This work was a proof-of-principle study of this in vivo noninvasive biodosimeter with β-emitting therapeutic radiopharmaceuticals. **Methods:** Six cell lines were exposed to external-beam radiotherapy (EBRT) or ^177^Lu-DOTATATE, after which the number of γH2AX foci and the clonogenic survival were measured. Mice bearing CA20948 somatostatin receptor–positive tumor xenografts were treated with ^177^Lu-DOTATATE or sham-treated and coinjected with ^111^In-anti-γH2AX-TAT, ^111^In-IgG-TAT control, or vehicle. **Results:** Clonogenic survival after external-beam radiotherapy was cell-line–specific, indicating varying levels of intrinsic radiosensitivity. Regarding in vitro cell lines treated with ^177^Lu-DOTATATE, clonogenic survival decreased and γH2AX foci increased for cells expressing high levels of somatostatin receptor subtype 2. Ex vivo measurements revealed a partial correlation between ^177^Lu-DOTATATE uptake and γH2AX focus induction between different regions of CA20948 xenograft tumors, suggesting that different parts of the tumor may react differentially to ^177^Lu-DOTATATE irradiation. **Conclusion:** ^111^In-anti-γH2AX-TAT allows monitoring of DNA damage after ^177^Lu-DOTATATE therapy and reveals heterogeneous damage responses.

Neuroendocrine tumors (NETs) comprise a heterogeneous group of neoplasms derived from peptide- and amine-producing cells of the neuroendocrine system. Despite their relatively low incidence, NETs are a heterogeneous and complicated tumor family and represent a significant clinical challenge requiring multidisciplinary care (1). Somatostatin receptor (subtype 2 or 5) expression in most differentiated neuroendocrine cancers allows treatment with somatostatin analogs such as octreotide, as well as imaging and therapy with radiolabeled somatostatin analogs. Compounds such as DOTATOC or DOTATATE are radiolabeled with γ-emitting radionuclides such as ^111^In for SPECT imaging, positron emitters such as ^68^Ga for PET imaging, or the β-emitting ^177^Lu or ^90^Y for molecular radiotherapy (MRT). MRT with small radiolabeled peptides, also called peptide receptor radionuclide therapy, using these β-emitting radiopharmaceuticals is now used routinely to treat NET patients (2). A large phase 3 study (the NETTER trial) demonstrated that ^177^Lu-DOTATATE significantly improved progression-free survival when compared with high-dose octreotide in patients with advanced midgut NETs, with minimal and transient side effects (3,4).

The radiobiologic aspects of ^177^Lu-DOTATATE, as for other MRT radiopharmaceuticals, have been underexplored (5). Despite its frequent and successful use, dosimetry is not always considered at the time that peptide receptor radionuclide therapy is planned or delivered. Little radiobiologic evaluation is performed (6,7), and therapy outcome is not measured until late (3 mo) after treatment, with no measurements of intratumoral heterogeneity, intracellular dosimetry, or short-term efficacy readouts. Although ^177^Lu-DOTATATE, ^90^Y-DOTATOC, and, increasingly, ^177^Lu-PSMA (prostate-specific membrane antigen) are widely used throughout Europe, therapy invariably consists of 2 or 4 intravenous administrations of 7.4 GBq, separated by 9–12 wk, mostly regardless of the patient’s size and weight, the extent of positive ^111^In-octreotide or ^68^Ga-DOTATATE uptake (measured by SPECT or PET imaging, respectively), or the inherent radiosensitivity of the tumor or patient (8,9). Importantly, most MRT dosimetry and radiobiology have been based on external-beam radiotherapy (EBRT) data because of a paucity of radiobiologic data on radionuclide therapy (10). This substitution of EBRT for MRT dosimetry cannot adequately account for the distinct and complex cellular localization of ionizing radiation with MRT and the distinctly different dynamic biologic response across the time frame of exposure during MRT. Although in vitro dosimetry methods exist, they require optimization for each cell line and are progressively complicated for each cell line with in vitro 3-dimensional spheroid cellular constructs (11). Even despite the best assessments of physical dose deposition, the effect that matters most is radiation cytotoxicity, and different cells, including cancer cells, react differently to the same absorbed dose. Thus, a biologic dosimetry approach may be used by measuring the extent of biologic response to ionizing radiation, such as DNA damage repair signaling. This biodosimetry approach can be considered a more direct measure of effective biologic dose and may have greater translational potential in the clinic.

The major cytotoxic effect of MRT is mediated by causing DNA damage. The β-decay of ^177^Lu-DOTATATE induces a variety of DNA damage, including single-strand breaks, as well as DNA double-strand break damage, one of the most lethal types of DNA damage. One of the responses to DNA double-strand break damage is phosphorylation of the histone isoform H2AX on serine-139 to form γH2AX. This phosphorylation is expressed in foci of several thousand copies around the DNA double-strand break site, where it acts as a scaffold to attract downstream DNA repair factors. γH2AX repair foci have traditionally been used in radiobiology to gauge the extent of DNA double-strand break damage after ionizing radiation, such as EBRT.

Previously, we have developed a radiolabeled modified version of an anti-γH2AX antibody, ^111^In-anti-γH2AX-TAT, that allows us to noninvasively visualize and quantify γH2AX expression in tumor tissue as a surrogate imaging-based measure of the extent of DNA double-strand break damage. The radiolabeled full-length antibody is modified with the TAT peptide, a cell-penetrating peptide that incorporates a nuclear localization sequence to enable the antibody to enter cells, penetrate the nucleus, and access its exclusively intranuclear target, γH2AX (12). We showed that ^111^In-anti-γH2AX-TAT, using SPECT imaging, enables measurement of DNA damage in several scenarios: after EBRT (13–15); after EBRT plus a radiosensitizer, such as an ATR inhibitor (16); after chemotherapies such as bleomycin, 5-FU, gemcitabine, or capecitabine in mouse models of breast or pancreatic cancer (13,17); and after DNA damage repair hyperactivation during tumorigenesis in a mouse model of HER2-driven breast cancer (12,13,18). In addition, we reported on a ^89^Zr-labeled version for PET imaging of γH2AX (15).

Apart from β-particles, ^177^Lu emits γ-rays (113 and 208 keV) that can be used for SPECT imaging. These can be applied to determine the accumulation of ^177^Lu in tissue and calculate the absorbed radiation dose. The γ-emissions of ^111^In do not overlap with ^177^Lu (171 and 245 keV), allowing dual-isotope imaging to simultaneously assess the physical dose distribution of ^177^Lu, as well as its biologic effect on DNA damage repair signaling, with ^111^In-anti-γH2AX-TAT. This method may therefore allow adaptive clinical treatment regimens.

Here, we demonstrate that ^177^Lu-DOTATATE therapy results in the formation of γH2AX foci in a mouse model of neuroendocrine cancer, allowing us to gauge the extent of DNA damage using the in vivo biodosimeter, ^111^In-anti-γH2AX-TAT, with dual-isotope SPECT imaging of ^177^Lu and ^111^In.

## MATERIALS AND METHODS

Full materials and methods are presented in the supplemental materials accompanying this article (supplemental materials are available at http://jnm.snmjournals.org) (19–24).

### General

^177^Lu-DOTATATE was prepared using previously described methods (25). Carrier-free ^177^Lu was obtained from ITG, and DOTATATE precursor was obtained from Cambridge Biosciences. ^177^Lu-DOTATATE was prepared to a molar activity of 50 MBq/nmol for in vitro use and 86 MBq/nmol (60 MBq/μg) for in vivo experiments, unless otherwise stated. The radiolabeling yield was routinely greater than 99.5%, as determined by instant thin-layer chromatography. Immunoconjugate was prepared and ^111^In-anti-γH2AX-TAT and ^111^In-IgG-TAT radiosynthesized using mouse monoclonal anti-γH2AX antibodies (clone JBW-301; Merck) or isotype-matched mouse nonspecific antibodies, as previously described (13).

### Cells, Cell Uptake, and Fractionation

Cell membrane association, internalization, and nuclear localization of ^177^Lu-DOTATATE were studied in the CA20948, BON1, QGP1, H727, U2OS, and U2OS^SSTR2^ cell lines. We used the rat pancreatic cancer cell line CA20948 for most of the work described here, including in vivo studies, since it is one of only a handful of pancreatic cancer models described in the literature that mimic the somatostatin overexpression found in many human NETs and form tumors in vivo. The cell line was derived from a rat pancreas and is acinar in origin yet displays a neuroendocrine phenotype. Cells were harvested using Accutase (Biolegend). Aliquots of 2 × 10^5^ cells in 200 μL of growth medium were exposed to ^177^Lu-DOTATATE (2.5 MBq/mL, 50 MBq/nmol) for increasing durations at 37°C for up to 24 h. The amount of ^177^Lu associated with cell membrane, cytoplasm, and nucleus was then measured using an automated γ-counter after cell fractionation as previously described (26).

### Clonogenic Survival

Cell suspensions (0.2 × 10^5^ cells) were prepared using Accutase, resuspended in growth medium (200 μL), and either treated with radiolabeled ^177^Lu-DOTATATE (0–2.5 MBq/mL, 50 MBq/nmol) and incubated at 37°C for 2 h, or exposed to external γ-irradiation (0–10 Gy, 1 Gy/min, using a ^137^Cs irradiator), or sham-treated. An aliquot of cells for each treatment condition was plated in 6-well plates with 2 mL of growth medium and incubated at 37°C in 5% CO_2_. After 2 wk, the number of colonies with more than 50 cells was counted to determine the clonogenic survival fraction. Geometries derived from confocal microscopy measurements of the dimensions of all cells in the panel allowed the calculation of S values, which were used for microdosimetry of ^177^Lu. The total absorbed radiation dose from ^177^Lu to cell nuclei was determined using a MIRD-based approach, assuming homogeneous ^177^Lu uptake on membrane, in cytoplasm, and in the nucleus. The total dose was calculated as the sum of self-dose and cross-dose.

### γH2AX Imaging by Confocal Microscopy

Cells were grown in 8-well culture chambers. After exposure of cells either to ^177^Lu-DOTATATE (2.5 MBq/mL, 50 MBq/nmol) for 2 h or to external-beam irradiation (6 Gy), they were left to recover in fresh growth medium for 1, 24, 48, or 72 h. Cells were then washed, fixed, permeabilized, and stained using a mouse anti-γH2AX antibody (clone JBW-301, 1:800).

### In Vivo Imaging

All animal procedures were performed in accordance with the U.K. Animals (Scientific Procedures) Act of 1986 and with local ethical committee approval. Female athymic nude mice were housed in individually ventilated cages in groups of up to 5 per cage in a facility with an artificial day–night cycle and ad libitum access to food and water. Tumor xenografts were generated by subcutaneous injection of cell suspensions (10^6^ cells in 100 μL of serum-free growth medium) in the right hind flank. Static SPECT/CT images were acquired at 1, 24, 48, and 72 h after an intravenous bolus administration of ^177^Lu-DOTATATE (20 MBq, 0.33 μg, in 100 μL of phosphate-buffered saline). In a separate study, immediately after the 1-h SPECT image the mice were additionally administered an intravenous bolus of ^111^In-anti-γH2AX-TAT, ^111^In-IgG-TAT (5 MBq, 5 μg, in 100 μL of phosphate-buffered saline), or phosphate-buffered saline control (Supplemental Fig. 1). The average tumor size at the start of the study was 177 ± 101 mm^3^. The average weight of the animals was 18 ± 1.1 g. SPECT/CT images were acquired in list mode for approximately 10 min using a single-gantry SPECT/CT and PET/CT scanner (VECTor^4^CT; MILabs) equipped with a high-energy ultra-high-resolution rat and mouse collimator containing pinhole apertures of 1.8-mm diameter. Reconstructed images were viewed and analyzed using PMOD (version 3.38; PMOD Technologies). Five animals were used per group. After the final imaging session, the animals were culled, and blood and selected tissues were harvested. ^177^Lu quantification on SPECT images was based on an analysis of a series of standards with known activity. Dual-isotope image reconstruction and quantification was performed using a series of phantoms containing a range of ^111^In:^177^Lu mixtures (Supplemental Fig. 2). Digital autoradiography and immunofluorescence confocal microscopy staining for γH2AX was performed on 10-μm tumor sections. U2OS or U2OS^sstr2^ cells did not form xenografts in BALB/c *nu/nu* mice in our hands. The absorbed radiation dose from ^177^Lu was calculated as previously described, based on volume-of-interest–derived volume measurements (3). The absorbed dose and absorbed dose rates were calculated at each time point using the sphere model features in the IDAC-Dose2.1 code for lymphoid tissue at a 1.03 g/mL density.

### Statistical Analysis

All statistical and regression analyses were performed using Prism (version 7; GraphPad Software). Linear regression with runs testing was used to check for correlations between measurements. After testing for normality using a Shapiro–Wilk test, means were compared using a *t* test with Welch correction for nonequal variances, when applicable. One-way ANOVA followed by Dunnet posttesting was used to compare multiple groups. Two-way ANOVA was used to analyze grouped data. All results are reported as the mean ± SD for at least 3 independent replicates.

## RESULTS

### ^177^Lu-DOTATATE Exposure and EBRT Cause Differential Effects in a Set of Cell Lines In Vitro

**FIGURE 1.**
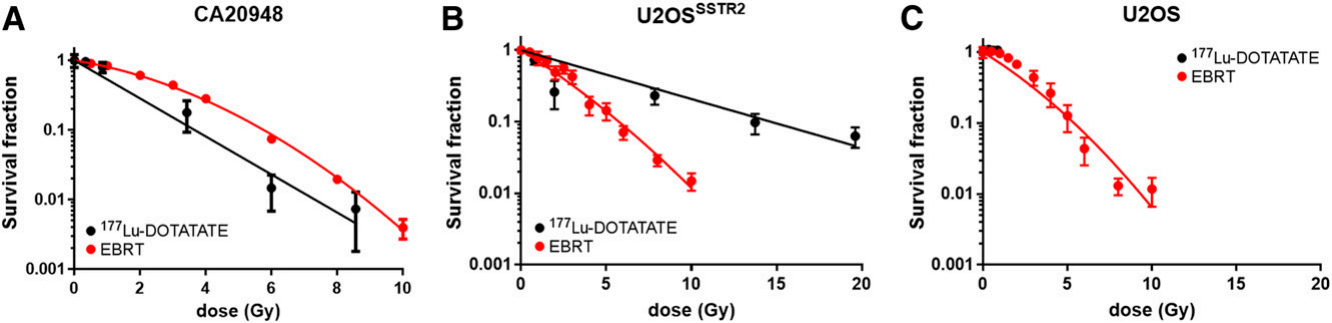
Clonogenic survival after in vitro exposure of cancer cell lines to varying amounts of ^177^Lu-DOTATATE or increasing amounts of EBRT: CA20948 cells (A), U2OS^sstr2^ cells (B), and wild-type U2OS cells (C). Absorbed radiation doses for ^177^Lu were based on ^177^Lu uptake data obtained separately (Supplemental Fig. 4).

Clonogenic survival after EBRT (0–10 Gy) in a panel of 6 cell lines revealed that all 6 lines present with inherently distinct radiation sensitivities (Fig. 1; Supplemental Fig. 3; Supplemental Table 1), apart from the U2OS/U2OS^sstr2^ pair, for which transfection of somatostatin receptor subtype 2 has no significant effect on clonogenic survival (*P* > 0.05). D90 values (the absorbed radiation dose at which clonogenic survival has dropped 10-fold) are 5.3, 5.4, 5.5, 5.7, 8.0, and 9.5 Gy for U2OS, U2OS^sstr2^, BON1, CA20984, H727, and QGP1 cells, respectively, indicating that the various cells have varying levels of sensitivity to EBRT.

**FIGURE 2.**
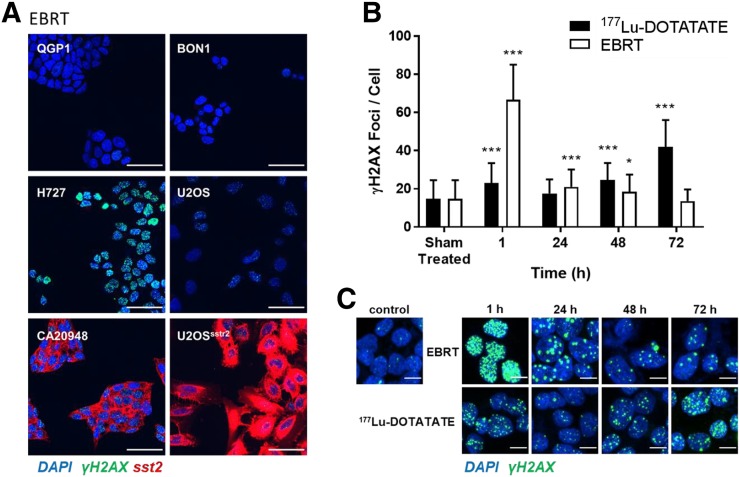
γH2AX focus formation in panel of cell lines. (A) Cells were stained for γH2AX (green) and somatostatin receptor subtype 2 (red) 1 h after exposure to 4 Gy of EBRT. 4′,6-diamidino-2-phenylindole (DAPI) was used to stain cell nuclei (blue) (scale bar = 50 μm). (B) Number of γH2AX foci per cell was determined at various intervals after exposure of CA20948 cells to ^177^Lu-DOTATATE for 2 h or after EBRT (6 Gy). *P < 0.01. ***P < 0.0001. (C) Representative immunocytochemistry micrographs (γH2AX = green, nuclei = blue) (scale bar = 10 μm).

Uptake of ^177^Lu-DOTATATE in a panel of 6 cancer cell lines in vitro occurred in line with expression of somatostatin receptor subtype 2 and resulted in reduced clonogenic survival in cell lines expressing somatostatin (Figs. 1–2; Supplemental Fig. 4). Transfection of somatostatin-negative U2OS cells to stably express somatostatin receptor subtype 2 receptors resulted in a 40-fold increase in cell-associated ^177^Lu after 2 h of exposure to ^177^Lu-DOTATATE (6.2 ± 1.7 vs. 250 ± 1.6 mBq/cell; *P* < 0.0001) (Supplemental Fig. 4). CA20948 cells, which naturally express high levels of somatostatin receptor subtype 2, when exposed to ^177^Lu-DOTATATE took up ^177^Lu (57 ± 5.0 mBq/cell), in contrast to QGP1, BON1, or H727 cells, which all express low levels of somatostatin receptors (8.9 ± 2.3, 6.2 ± 5.4, and 8.4 ± 1.1 mBq/cell, respectively). Not surprisingly, clonogenic survival was reduced significantly only in cells that express somatostatin and thus take up ^177^Lu-DOTATATE.

The amount of ^177^Lu associated with the membrane, cytoplasm, and nucleus of all cells at various times after exposure to ^177^Lu-DOTATATE (Supplemental Fig. 4) was determined from cellular fractionation. Although most cell-associated ^177^Lu was associated with the membrane at all time points, a significant amount was associated with the cytoplasmic fraction (13% in CA20948 cells at 2 h) but very little in the nucleus (<0.1%). Differences from previously reported results may be explained by the fact that, here, we performed the measurements not on adherent cells but on cells in suspension. Given the range of β-particles emitted by ^177^Lu (on average, 1.7 mm), this method results in a radiation dose to the cells and their nuclei, resulting in reduced clonogenic survival.

Within these monoclonal cell cultures, clonogenic survival of cells after exposure to ^177^Lu-DOTATATE correlated well with absorbed dose (Fig. 1). However, comparing CA20948 and U2OS^sstr2^ cells, the same absorbed radiation dose from ^177^Lu-DOTATATE resulted in clonogenic survival different from that after EBRT. Clonogenic survival in CA20948 cells was higher for EBRT than for the same radiation dose of ^177^Lu-DOTATATE (*P* < 0.0001), whereas in U2OS^sstr2^ cells it was lower (*P* < 0.0001) (Supplemental Table 1). This finding reinforces previous reports that the same dose of EBRT and MRT does not result in the same biologic effect and that this difference may vary among cell lines (27).

### ^177^Lu-DOTATATE Exposure in Somatostatin-Positive Cells Results in γH2AX Foci In Vitro

Exposure of all cells to EBRT led to formation of γH2AX foci, to different extents in each cell type (Fig. 2A). In CA20498 cells, exposure to ^177^Lu-DOTATATE for 2 h also resulted in DNA double-strand break damage, as measured by γH2AX foci (Figs. 2B and 2C). Interestingly, the number of γH2AX foci per cell continued to increase significantly for up to 72 h after exposure to ^177^Lu-DOTATATE (42 ± 14 vs. 15 ± 9.7 in treated vs. nontreated cells; *P* < 0.0001). This finding was in stark contrast to the number of γH2AX foci for a single dose of EBRT, after which γH2AX foci were high shortly after irradiation (67 ± 18; *P* < 0.0001) but soon returned to pretreatment levels (13 ± 6.1; *P* > 0.05 at 72 h), as is expected in most cells without DNA damage repair defects.

A similar result was obtained in U2OS^sstr2^ cells, although here the number of γH2AX foci did not increase at 72 h after exposure to ^177^Lu-DOTATATE but at all times was higher than the number in wild-type U2OS cells (*P* < 0.0001; Supplemental Fig. 5). These results agree with earlier results from Dalm et al., who showed the formation of another type of DNA damage repair foci, 53BP1 foci, after ^177^Lu-DOTATATE treatment of U2OS^sstr2^ cells (28,29).

Thus, DNA damage repair signaling as measured by γH2AX foci after exposure to ^177^Lu-DOTATATE is distinct from that after EBRT.

### ^177^Lu-DOTATATE Uptake in Xenograft Tumors Induces γH2AX Foci In Vivo

**FIGURE 3.**
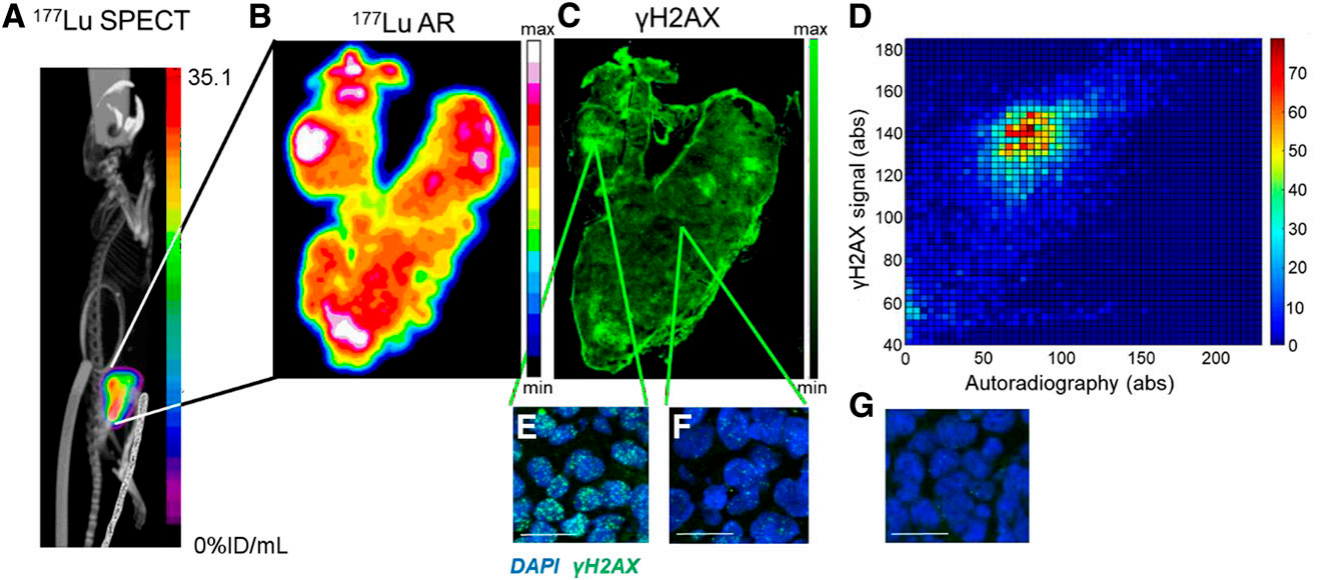
(A) Representative SPECT/CT image 72 h after intravenous administration of ^177^Lu-DOTATATE (20 MBq, 0.33 μg) in CA20948 xenograft–bearing athymic mouse. (B) Autoradiography (AR) performed on tumor section harvested from same mouse. (C) Adjacent section was stained for γH2AX, and resulting fluorescence micrograph was coregistered to AR image (scale bar = 800 μm) (D) Density scatterplot based on pixel-by-pixel analysis of γH2AX signal vs. autoradiography (omitting edge effects on immunohistochemistry). (E and F) High-resolution details of immunohistochemistry in C, demonstrating γH2AX foci in areas of intense or minimal staining (γH2AX = green; nuclei = blue; scale bar = 20 μm). (G) Immunohistochemistry for γH2AX on representative tumor section from mouse that was treated with vehicle control only.

Intravenous administration of ^177^Lu-DOTATATE to CA20948 xenograft–bearing mice resulted in high tumor uptake (36 ± 4.5 percentage injected dose [%ID]/mL at 24 h after administration; Fig. 3A), whereas other xenografts took up far less ^177^Lu-DOTATATE (*P* < 0.0001), in line with in vitro results and somatostatin expression levels (Supplemental Fig. 6A). Dynamic SPECT imaging revealed that maximum tumor uptake in CA20948 xenografts was reached at 60 min after administration (Supplemental Fig. 6B).

Comparable to our in vitro results, high ^177^Lu-DOTATATE uptake and γH2AX focus formation was observed in CA20948 xenografts 72 h after administration of ^177^Lu-DOTATATE (Figs. 3B–3F), compared with nontreated tumors (Fig. 3G). The delivery of ^177^Lu-DOTATATE to the tumors was heterogeneously distributed, as has been observed previously (30). A comparison of autoradiography showing ^177^Lu uptake in a tumor section with immunohistochemistry staining for γH2AX revealed that, in general, areas of tumor with higher ^177^Lu uptake showed a higher number of γH2AX foci per cell (Fig. 3E) and areas with lower ^177^Lu uptake showed fewer γH2AX foci per cell (Fig. 3F), but this correlation was not linear or significant. Only a few cells with pan-nuclear staining, indicating late-stage apoptosis, were observed. Interestingly, a large number of regions with intermediate ^177^Lu uptake could also be observed, with the number of γH2AX foci being highly variable. A correlation plot quantitatively comparing the 2 signals revealed a similar lack of pattern (Fig. 3D). Similar observations were made for all tumors (3 additional examples are shown in Supplemental Fig. 7), indicating that yH2AX may be used as a marker for the biologic effect of ^177^Lu therapy.

### ^111^In-Anti-γH2AX-TAT Allows In Vivo Imaging of DNA Damage After ^177^Lu-DOTATATE Therapy

^111^In-anti-γH2AX-TAT enabled imaging of γH2AX in vivo. Dual-isotope imaging of ^177^Lu-DOTATATE and ^111^In-anti-γH2AX-TAT allowed concurrent imaging of tumor-associated ^177^Lu and visualization of the DNA double-strand break damage resulting from the emitted β-particles. The ability of the VECTor^4^ imaging system to simultaneously acquire images for ^111^In and ^177^Lu was evaluated using phantoms containing mixtures of known amounts of either radionuclide. Samples containing only ^177^Lu did not show any signal in the reconstructed ^111^In image, and vice versa. Importantly, quantification of ^111^In or ^177^Lu was not influenced by the presence of the other isotope (*R* = 0.99, *P* < 0.0001; Supplemental Fig. 2), corroborating earlier reports on dual-isotope imaging with this system (31,32).

**FIGURE 4.**
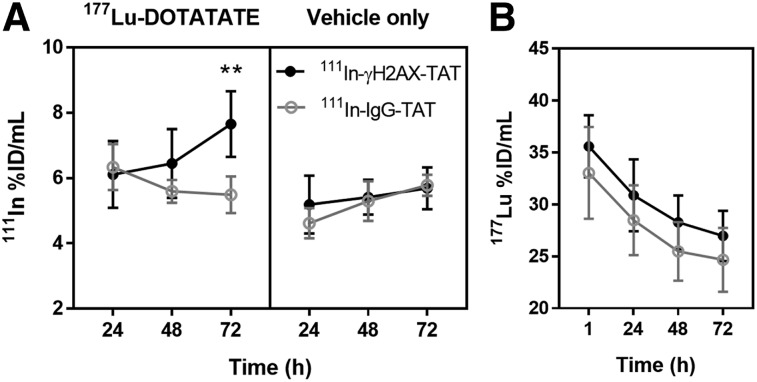
(A) Tumor uptake of ^111^In-anti-γH2AX-TAT or ^111^In-IgG-TAT at various times after treatment of CA20948-bearing mice with ^177^Lu-DOTATATE (20 MBq, 0.33 μg) or vehicle control. **P < 0.005. (B) Uptake of ^177^Lu in tumor of ^177^Lu-DOTATATE–treated animals.

**FIGURE 5.**
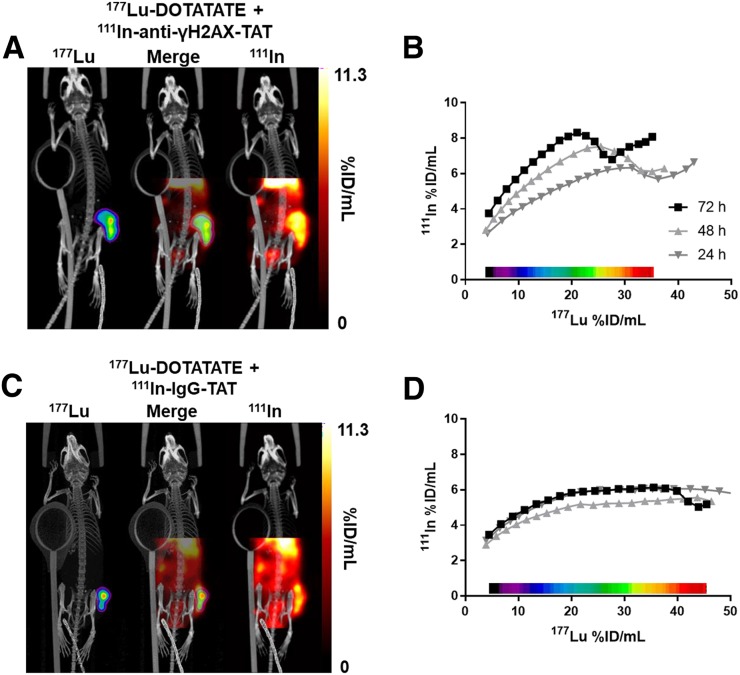
(A) Representative dual-isotope SPECT/CT images of mice 71 h after intravenous administration of ^111^In-anti-γH2AX-TAT (5 MBq, 5 μg) and 72 h after intravenous administration of ^177^Lu-DOTATATE (20 MBq, 0.33 μg). Tumor is indicated by purple contour in ^177^Lu image. (B) Correlation between ^111^In and ^177^Lu signal in tumor volume in voxel collections based on ^177^Lu signal quantification in SPECT image of animal in A. (C) Representative dual-isotope SPECT/CT images of mice after administration of ^111^In-IgG-TAT (5 MBq, 5 μg) and ^177^Lu-DOTATATE (20 MBq, 0.33 μg). Tumor is indicated by purple contour in ^177^Lu image. (D) Correlation between ^111^In and ^177^Lu signal in tumor volume in voxel collections based on ^177^Lu signal quantification in SPECT image of animal in C.

^111^In-anti-γH2AX-TAT uptake increased in tumors treated with ^177^Lu-DOTATATE. Volume-of-interest analysis of the ^111^In signal in SPECT/CT images acquired at various time points revealed a significant increase in tumor uptake of ^111^In-anti-γH2AX-TAT in CA20948 tumor xenografts 72 h after injection (73 h after intravenous administration of 20 MBq of ^177^Lu-DOTATATE), as compared with ^111^In-anti-γH2AX-TAT uptake in control animals (*P* = 0.0033) or uptake of the nonspecific control compound, ^111^In-IgG-TAT, with or without ^177^Lu treatment (*P* < 0.0001) (Figs. 4 and 5). Uptake of the nonspecific control compound, ^111^In-IgG-TAT, was not altered by treatment of the tumors with ^177^Lu-DOTATATE (*P* = 0.41), confirming that the effect on ^111^In-anti-γH2AX-TAT is not due to physiologic changes that may affect nonspecific uptake of the IgG-TAT construct. Detailed data on the tumor uptake in each mouse are reported in Supplemental Figure 8. In addition, we observed no significant differences on the uptake of ^177^Lu in tumors or any normal tissues after administration of ^111^In-anti-γH2AX-TAT compared with ^111^In-IgG-TAT (*P* > 0.05) (Fig. 4B; Supplemental Fig. 9). In vivo tumor uptake of ^111^In-anti-γH2AX-TAT followed the same trend over 72 h as the number of γH2AX foci in vitro after brief exposure to ^177^Lu-DOTATATE.

No statistically significant differences in uptake of ^111^In-anti-γH2AX-TAT or ^111^In-IgG-TAT were observed in any organ of mice exposed to ^177^Lu-DOTATATE versus untreated animals (*P* > 0.05), with the exception of the spleen (*P* = 0.0009) (Supplemental Fig. 9). Given the very low uptake of ^177^Lu in the mouse spleen (0.43 ± 0.23 %ID/g at 72 h), radiation exposure seems an unlikely source in mice, although in humans the spleen receives a nonnegligible dose after ^177^Lu-DOTATATE (4.5–15 Gy over 2–5 cycles) (33). Notably, in our experimental setup, we observed no differences in uptake of ^111^In-anti-γH2AX-TAT in mouse kidney, the tissue that is the most exposed to ^177^Lu radiation, second only to tumor.

Volume-of-interest analysis of the ^177^Lu signal in all images allowed us to calculate the average absorbed radiation dose to the tumor in ^177^Lu-DOTATATE–treated animals as 12.9 ± 3.4 Gy (after 72 h; Supplemental Table 2; Supplemental Fig. 10), similar to previously reported values (29). Clearance from the tumor xenografts occurred with a mean effective half-life of 46.3 ± 8.6 h. There was no statistical difference in the average absorbed dose from ^177^Lu between animals imaged with ^111^In-anti-γH2AX-TAT and animals imaged with ^111^In-IgG-TAT (*P* = 0.15, Mann–Whitney test). Contrary to our earlier observations after EBRT (13), the accumulated absorbed dose from ^177^Lu, and the dose rate of ^177^Lu at any given time, did not correlate with ^111^In-anti-γH2AX-TAT uptake in the tumor, at least not in the limited dataset analyzed here (*P* > 0.55, *n* = 5).

### ^111^In-Anti-γH2AX-TAT Shows Heterogeneity In Vivo

Pixel-by-pixel segmentation of tumor volumes, based on the magnitude of the ^177^Lu signal, allowed correlation with the amount of ^111^In signal in various substructures within the tumor (Fig. 5). Consistent with our earlier ex vivo γH2AX focus measurements (Fig. 3), qualitative analysis revealed that, in general, areas within the tumor with higher ^177^Lu uptake also took up more ^111^In-anti-γH2AX-TAT at all time points, a correlation that was linear up to approximately 20 %ID/g of ^177^Lu (*R*^2^ = 0.9843, *P* < 0.0001, Fig. 5B), but the same was not true for ^111^In-IgG-TAT (Fig. 5D). However, consistent with our earlier ex vivo γH2AX focus measurements (Fig. 3), our results hint toward a more complex relationship between ^177^Lu uptake and the radiobiologic response, especially at the higher end of ^177^Lu exposure, than would be suggested by a ^177^Lu-radiation–deposited dose alone.

## DISCUSSION

Here we show, for the first time to our knowledge, that the DNA double-strand break damage marker γH2AX, as induced by MRT with ^177^Lu, can be visualized and quantified noninvasively by whole-body molecular imaging. First, we confirmed that exposure of somatostatin-expressing cells to ^177^Lu-DOTATATE in vitro resulted in reduced clonogenic survival. Different cell lines responded differently to the same absorbed ^177^Lu dose. The same was true for EBRT. Nonetheless, sensitivity to EBRT did not correlate linearly with sensitivity to ^177^Lu. DNA double-strand break damage was observed in vitro by immunofluorescence, as measured by γH2AX foci. The kinetics of γH2AX formation and dissolution after ^177^Lu exposure was different from that after EBRT. It has been shown previously that the therapeutic success of ionizing radiation correlates closely with the induction of DNA double-strand break damage, especially with late, unrepaired damage (34). ^177^Lu-DOTATATE causes DNA damage in vivo in tumor tissue and thus causes expression of γH2AX. We demonstrated that this induction of γH2AX after ^177^Lu-DOTATATE therapy can be monitored by SPECT imaging with ^111^In-anti-γH2AX-TAT. We were able to simultaneously study, in the whole tumor, the relationship between ^177^Lu distribution, as a surrogate for absorbed dose, and one aspect of the radiobiologic response of the tumor, DNA double-strand break damage repair, as measured by γH2AX expression. On average over the whole tumor, ^111^In-anti-γH2AX-TAT uptake is increased after ^177^Lu-DOTATATE therapy over 72 h, similar to our in vitro immunofluorescence results. Most interesting, however, is that within each tumor, the amount of DNA damage as measured by γH2AX foci does not strictly correlate with the amount of ^177^Lu deposition within tumors (Figs. 3 and 5). This finding suggests a more complex relationship between the amount of ^177^Lu uptake and the macroscopic radiation dose deposited in various parts of the tumor, with the resulting biologic effects such as DNA damage repair.

This proof-of-principle study showed that DNA damage from MRT can be measured noninvasively and may potentially be used as an in vivo biodosimeter. To the best of our knowledge, this was the first study of its kind—one that measures the direct, mechanistic, biologic effects of MRT. Understandably, some challenges need to be overcome before translation to the clinic is possible. Our initial results here were obtained using athymic mice bearing rat xenografts, but the results can be readily extrapolated to the human situation, given that similar interplay exists between ^177^Lu uptake, heterogeneous ^177^Lu tumor uptake, and DNA damage and repair. Without underestimating the importance of the physical radiation dose deposited in tumor and normal tissue for all MRT agents, the radiobiologic effects of MRT need to be considered when predicting therapeutic outcome. Different tumors react differently to EBRT, as demonstrated in the limited panel of 6 tumor cell lines. The cell line panel used here also portrayed differences in γH2AX kinetics after EBRT, given their inherent differences in radiosensitivity and potential further dissimilarities in cell signaling due to mutations, epigenetic or posttranslational variations in DNA damage repair proteins, and differential stress responses. Therefore, the same must be true for MRT. In addition, MRT effects will be complicated by the combination of receptor expression level, radionuclide uptake, radionuclide deposited dose, intratumoral heterogeneity (11), subcellular distribution (35), and radiobiologic effects, as well as tumor microenvironmental parameters such as hypoxia and systemwide effects such as immune-system effects. Here, we have not considered the effects of those systemwide consequences. As used here, ^111^In-anti-γH2AX-TAT provides one potential biodosimeter to establish a measurement of the radiobiologic effects of MRT with ^177^Lu-DOTATATE. Its clinical applicability is yet to be tested. It is worth noting that γH2AX, and therefore imaging with ^111^In-anti-γH2AX-TAT, remains a secondary biomarker, and γH2AX can also be upregulated as a result of some other cellular stress responses, such as oncogenic stress, increased genomic instability, and late-stage apoptosis (18,36). Therefore, ^111^In-anti-γH2AX-TAT imaging may not reflect DNA double-strand break damage only. The most likely alternative cause of γH2AX upregulation is MRT-induced apoptosis, resulting in pan-nuclear γH2AX staining. However, we did not observe this in the time span during which we imaged γH2AX here, making ^111^In-anti-γH2AX-TAT a suitable agent for imaging the early DNA damage response.

In this work, we showed imaging of DNA damage after ^177^Lu therapy based on a DOTATATE vector. However, the same system can be used to evaluate other MRT agents, such as ^177^Lu-PSMA, which is increasingly applied for the treatment of prostate cancer, long-range β-emitting radiopharmaceuticals based on ^90^Y or ^131^I, or targeted α-emitter therapy based on ^225^Ac or ^231^Bi, given their propensity to cause complex DNA damage and abundant γH2AX signals (37). γH2AX has also been suggested as a biomarker of normal-tissue toxicity, such as renal toxicity after MRT (38), and a marker of peripheral blood lymphocyte toxicity (39). However, we did not observe any significant changes in renal uptake of ^111^In-anti-γH2AX-TAT, likely because the amount of ^177^Lu-DOTATATE used in our studies did not cause clinically significant renal damage or because the physiologic renal uptake of ^111^In-anti-γH2AX-TAT (5.1 ± 0.4 %ID/g at 72 h after administration in animals not exposed to ^177^Lu-DOTATATE) prevents observation of these differences.

Agents that image response to therapy, such as ^111^In-anti-γH2AX-TAT or its PET alternative, ^89^Zr-anti-γH2AX-TAT (15), might find applications in adaptive therapy. Similar to measuring the genotoxic effects of chemotherapy (17), EBRT (13), and radiosensitizers (16), measuring the effects of radionuclide therapy in vivo may allow adjustment of the therapeutic regimen in accordance with the individual patient’s response to that treatment. In addition, noninvasive imaging can reveal differential responses in multiple tumors in the same patient or elucidate the heterogeneous biologic response within the same tumor. Using therapeutic response assessment with molecular imaging, making rapid decisions becomes possible, rather than having to await the anatomic changes that potentially follow later, after successful therapy. It is notable, however, that metabolic responses to some of the latest targeted therapies are not necessarily accompanied by an anatomically obvious response (40). Such stratification, possible after a single cycle of MRT, allows for an adaptive treatment design (5), a dose reduction to avoid side effects, assessment of combination therapies, or, in the absence of any measurable response, initiation of palliative options designed toward improving quality of life. Moreover, this strategy may also be a financially prudent one, given the high cost of each dose of Lutathera ($47,500; Advanced Accelerator Applications).

## CONCLUSION

Imaging of the DNA damage response using ^111^In-anti-γH2AX-TAT provides unique insight after ^177^Lu-DOTATATE therapy and allows the visualization of biologic response. This includes not only intratumoral heterogeneity but also interlesion heterogeneity within the same patient.

## DISCLOSURE

Edward O’Neill, Nadia Falzone, Katherine Vallis, Samantha Terry, Julie Nonnekens, Marion de Jong, and Bart Cornelissen were supported by MRC (MR/P018661/1). Bart Cornelissen, Michael Mosley, Sean Smart, P. Danny Allen, and Veerle Kersemans were supported by CRUK through the CRUK/MRC Oxford Institute for Radiation Oncology. Julia Baguña Torres was supported by PCRF. Samantha Terry was also supported by the Academy of Medical Sciences [SBF001\1019] and the Wellcome/EPSRC Centre for Medical Engineering at King’s College London [WT 203148/Z/16/Z]. Julie Nonnekens was also supported by the Daniel den Hoed Foundation. Julie Nonnekens and Marion de Jong have received financial support for research projects from AAA. No other potential conflict of interest relevant to this article was reported.

**KEY POINTS**

**QUESTION:** Can the radiolabeled antibody ^111^In-anti-γH2AX-TAT be used in vivo to visualize and quantify the γH2AX foci generated at the sites of double-strand DNA breaks caused by ^177^Lu-DOTATATE therapy?
**PERTINENT FINDINGS:** The γH2AX foci induced by ^177^Lu-DOTATATE could be imaged by SPECT in vivo using ^111^In-anti-γH2AX-TAT, and they correlated with ex vivo and in vitro γH2AX levels. γH2AX expression revealed intratumoral and interlesion heterogeneity with the absorbed ^177^Lu dose, suggesting a complex biologic response to ^177^Lu therapy.
**IMPLICATIONS FOR PATIENT CARE:** ^111^In-anti-γH2AX-TAT can potentially be used as a biodosimeter for optimizing radionuclide treatments such as ^177^Lu-DOTATATE, both in preclinical investigations and in the design of personalized, adaptive treatment regimens for patients.
