# Supplementary material for: Imaging DNA Damage Repair In Vivo After 177Lu-DOTATATE Therapy
Source: J Nucl Med. 2020 May;61(5):743–50. doi: 10.2967/jnumed.119.232934 (PMC7198382; doi:10.2967/jnumed.119.232934)
Supplement: Supplementary file 1 [file jnm232934SupplementalData.pdf]

Imaging DNA Damage Repair *in vivo* Following ^{177}Lu-DOTATATE Therapy

Edward O'Neill[1], Veerle Kersemans[1], P. Danny Allen[1], Samantha Y.A. Terry[2], Julia Baguña Torres[1], Michael Mosley[1], Sean Smart[1], Boon Quan Lee[1], Nadia Falzone[1], Katherine A. Vallis[1], Mark W. Konijnenberg[3], Marion de Jong[3], Julie Nonnekens[3,4,5], Bart Cornelissen[1,*]

[1] CRUK/MRC Oxford Institute for Radiation Oncology, Department of Oncology, University of Oxford, Oxford, UK
[2] Department of Imaging Chemistry and Biology, King's College London, London, UK
[3] Department of Radiology and Nuclear Medicine, Erasmus MC, Rotterdam, The Netherlands
[4] Department of Molecular Genetics, Erasmus MC, Rotterdam, The Netherlands
[5] Oncode Institute, Erasmus MC, Rotterdam, The Netherlands

General

All reagents were purchased from Sigma-Aldrich unless otherwise stated and were used without further purification. The chelating agent *p*-SCN-Bn-DTPA was purchased from Macrocyclics Inc. (Dallas, TX). Water was deionised using a Barnstead NANOpure purification system (Thermo Scientific) and had a resistance of >18.2 MΩ/cm at 25°C. Protein concentration measurements were made using a ND-1000 spectrophotometer (NanoDrop Technologies, Inc.). Instant thin-layer chromatography (iTLC) was performed with glass microfiber chromatography paper (Agilent Technologies) and strips were analysed with a Bioscan AR-2000 radio-TLC scanner (Eckert & Ziegler). pH was determined using pH indicator paper (Merck Millipore). Radioactivity measurements were made using a CRC-25R dose calibrator (Capintec, Inc.).

Cells

Six cancer cell lines were used, incorporating cells with high and low expression levels of SST2, and to compensate for possible defects in DNA damage response signaling in one of the cell lines. The neuroendocrine cancer patient-derived BON1, QGP1, and H727 cells express low levels of SST2. BON1 (derived from a human enterochromaffin cell serotonin-producing pancreatic neuroendocrine tumor) was kindly provided by Professor Simona Glasberg from Hadassah-Hebrew University Medical Center and maintained in 1:1 DMEM/Ham's F-12. QGP1 (human pancreatic somatostatinoma) was obtained from the Japanese Collection of Research Bioresources Cell Bank (JCRB) and maintained in RPMI 1640. H727 (human lung neuroendocrine carcinoma) obtained from the American Type Culture Collection was maintained in RPMI 1640. U2OS cells were obtained from ATCC, whereas U2OS cells transfected to stably express high SST2 levels (U2OS^{sstr2}) were generated at Erasmus MC (*1*). Both U2OS variants were maintained in DMEM. CA20948 cells, a rat pancreatic neuroendocrine cancer cell line that overexpresses SST2, were originally derived from solid tumor tissue at Erasmus MC, and maintained in Dulbecco's Modified Eagle Medium (DMEM) supplemented with 1 mM sodium pyruvate (*2*). All cells were supplemented with 10% fetal bovine serum (FBS), 2 mM L-glutamine, 100 units/mL penicillin, and 0.1 mg/mL streptomycin. Cells were grown in a 37°C environment containing 5% CO_2 and were harvested and passaged as required using trypsin-EDTA solution. Cells were authenticated by the provider and regularly tested for the absence of mycoplasma contamination. The cumulative length of culture was less than 6 months following retrieval from liquid nitrogen storage.

Cell uptake and cell fractionation

Cell membrane-association, internalization and nuclear localisation of ^{177}Lu-DOTATATE were studied in CA20948, BON1, QGP1, H727, U2OS and U2OS^{SSTR2} cell lines. Radiolabelled ^{177}Lu-DOTATATE (2.5 MBq/mL, 50 MBq/nmol) was added to aliquots of 2×10^5 cells, previously detached using Accutase (Biolegend Inc), in 200 µL of their respective growth media in 1.5 mL tubes at 37°C. The samples were gently agitated by a rocking plate inside the incubator. All samples were centrifuged at 500×g for 2 min, and culture medium was removed. The cells were resuspended in ice-cold PBS (pH 7.4) and centrifuged again. The PBS was removed, combined with the previously separated medium and counted in a γ-counter to measure cell-free radioactivity. Cell-surface bound material was removed by resuspension of the cells in 500 µL of glycine buffer (0.1 M glycine.HCl, pH 2.5) for 6 min on ice (*3*). Samples were centrifuged at 500×g for 2 min, the supernatant containing the membrane-bound fraction was removed, and cells washed twice with

PBS and combined with the fractions containing glycine buffer. Cell pellets were then resuspended in 500 µL of cell membrane lysis buffer (25 mM KCl, 5 mM $MgCl_2$, 10 mM Tris-HCl and 0.5% NP-40). After incubation on ice for 6 min, the lysate was centrifuged at 1000×g for 2 min to separate cytoplasm-associated ^{177}Lu (supernatant) from the nuclear-associated content. The nuclear fraction was washed with 500 µl of lysis buffer, the suspension was recentrifuged and the supernatant was removed and combined with that previously separated. Radioactivity in the membrane, cytoplasmic and nuclear fractions was measured using an automated gammacounter (*3,4*).

***In vitro* immunofluorescence**

Cells grown in 8-well chambered cell culture slides, 10,000 in a total volume of 200 µL cell culture medium (Falcon), were exposed to ^{177}Lu-DOTATATE (2.5 MBq/mL, 50 MBq/nmol) and incubated at 37°C in 5% CO_2 for 2 h; or exposed to external irradiation (6 Gy); or sham treated/irradiated. Cells were then washed with PBS, provided with fresh growth media, and left to recover for 1, 24, 48, or 72 h at 37°C in 5% CO_2. After recovery, cells were washed with PBS and fixed using 4% paraformaldehyde solution for 10 min. After washing, the cells were permeabilised using 0.2% triton X-100 in TBS buffer (50 mM Tris-Cl, pH 7.5. 150 mM NaCl) for 10 min at room temperature, and briefly rinsed with TBS. Non-specific binding was blocked with 2% BSA in TBST (0.1% Tween-20/TBS) for 1 h at room temperature. Primary antibody (mouse anti γH2AX Millipore, Clone JBW-301, 1:800) diluted in blocking buffer was applied directly on the slide and incubated overnight at 4°C, and then washed with TBST. Fluorescently labelled secondary antibody, dylight-488 goat anti-mouse (Invitrogen A11034, 1:250 in blocking buffer) was applied and incubated for 1 h at room temperature. After further washing with PBS, slides were mounted using Vectashield containing DAPI to visualise cell nuclei (Vector Labs). Images were acquired on an Andor Dragonfly microscope (Oxford Instruments), using a 40x/1.3 oil lens to generate a series of z-stacks.

***In vitro* Microdosimetry**

The total radiation absorbed dose from ^{177}Lu to cell nuclei was determined using a MIRD-based approach. The total dose was calculated as the sum of self-dose and cross-dose.

Cell dimensions: Determination of S-values for dosimetry depends on cell dimensions. These were measured by fluorescence microscopy. Live cells were detached using accutase (Biolegend), washed with PBS, incubated with Hoechst 33342 (Abcam) and CellBrite Orange cytoplasmic membrane dye (Insight Biotechnology) for 15 min at 37°C, then embedded in low melting point agarose (Trevigen), and deposited

on a microscope slide with a glass coverslip for immediate imaging. The average spherical dimensions of the cell membrane and nucleus for each cell type were calculated from the average of the length x width of the membrane and nucleus in the central plane of each suspended cell from z-stacks of fluorescence images collected using a Leica SP8 fluorescence microscope (Leica), using a 63x/1.4 oil lens.

Self-dose: Dose-point kernels (DPKs) were calculated with the general-purpose Monte Carlo code PENELOPE (*5*) using the complete electron spectrum of ^{117}Lu based on the unabridged β^- spectrum from MIRD RADTABS (*6*) and the spectrum of conversion, Auger and Coster-Kronig electrons from *BrIccEmis* (*7*), determined by means of event-by-event simulation as previously described (*8*). Photon dose was omitted due to the low intensity and small geometry. A total of $1\text{x}10^8$ primary particles were simulated in each run with a statistical uncertainty ≤ 0.4% achieved in each bin of a 1-nm-thick spherical water shell. Assuming a cell model of two concentric homogenous spheres of liquid water (mass density ρ = 1 gcm^{-3}), representing the cell and its nucleus and taking the nucleus as the target, absorbed fractions per decay (self-dose *S*-values) were calculated from the simulated DPKs to determine contributions from the nucleus (N←N), the cytoplasm (N←Cy) or the cell surface (N←CS).

Cross-dose: Two geometries representing cells in suspension (first 2 hours) and cells plated (the following 2 weeks) were considered. For the cells in suspension, a target cell is immersed in a spherical water medium of 2 mm in radius (33.5 µL). This particular radius is chosen as the continuous-slowing-down-approximation (CSDA) range of the most energetic β^- electrons ($E_{max,\beta-}$ = 498 keV) in water is about 1.7 mm. Radioactivity was assumed to be uniformly distributed throughout the water medium but outside the target cell. For the plated cells, a target cell is placed on the base and at the centre of a well of 1.75 cm radius. The well is filled with water of 2.08 mm depth (2 mL). In order to achieve similar levels of statistical uncertainty compared with the case of cells in suspension, a cuboid water medium of 4 mm in length and width and 2.08 mm in height (33.3 µL) surrounding the target cell was used as the source volume. A total of $1\text{x}10^8$ primary particles were simulated in each run. Cross-dose *S*-values was determined from the energy deposited in the target cell nucleus by the radioactivity distributed uniformly in the source volume.

Total self-dose to each cell was determined by the product of self-dose *S*-values and cumulative decays from different cell compartments calculated using trapezoidal integration for the first 2 hours using uptake data and assuming purely physical decays after that. Total cross-dose was similarly calculated by using cross-dose *S*-values and only considering cumulative decays from the environment as a result of physical decay.

SPECT/CT Imaging

SPECT/CT images were acquired using a VECTor^{4}CT scanner (MILabs, Utrecht, The Netherlands) equipped with a HE-UHR-RM collimator containing pinhole apertures of 1.8 mm diameter. The scanner was calibrated during each imaging session by imaging a phantom with either an indium-111 or lutetium-177 standard solution. Data were acquired in list mode (gamma spectrum collection from 0-1200 keV) using MILabs acquisition software v7.15. Triple-energy-window based scatter correction was applied for the indium-111 photon peaks (155.7-188 and 228-271.7 keV) and background peaks (148.8-155.7, 187.3-192.2, 225.4-228.3 and 271.7-282.6 keV) and lutetium-177 peaks (103-126 and 197-221 keV) and background peaks (98.4-103, 126-130.6, 192.2-197 and 221-225 keV). All images were reconstructed with MILabs reconstruction software v3.24 to 0.6 mm isotropic 3D voxel grids using dual matrix similarity regulated ordered-subset expectation maximisation (dual matrix SROSEM, using 4 iterations and 2 subsets for lutetium-177 and 4 subsets for indium-111). Subsequently, whole body CT images were acquired at 50 kV and 0.24 mA using continuous rotation (40 degrees/s). After reconstruction, the SPECT and corresponding CT data were co-registered and re-sampled to equivalent 200 µm isotropic voxels. CT-based attenuation correction was applied.

Reconstructed images were viewed and analysed using PMOD v.3.38 (PMOD Technologies, Zurich, Switzerland). Tumour uptake of indium-111 for lutetium-177 treated mice was calculated based upon VOIs generated by the segmentation tool in PMOD, which represented CT-based volumes that contained magnitudes equal and greater than 20%ID/mL of lutetium-177. . A spherical VOI with the same volume was used to calculate tumour uptake of indium-111 in mice that did not receive ^{177}Lu-DOTATATE. Results were reported as percentage of the injected dose per mL of tissue (%ID/mL).

***In Vivo* Studies**

All animal procedures were performed in accordance with the UK Animals (Scientific Procedures) Act 1986 and with local ethical committee approval. For ^{177}Lu-DOTATATE biodistribution studies, xenograft tumours were established in the right hind flank of female athymic BALB/c *nu/nu* mice (Charles River) by subcutaneous injection of CA20948 (5×10^6 cells), or QGP1 (1×10^7 cells), or BON1 (5×10^6 cells), or H727 (5×10^6 cells) in sterile PBS (100 µL). When tumours reached a diameter of approximately 10 mm, ^{177}Lu-DOTATATE (20 MBq, 0.33 µg) in sterile PBS (100 µL) was injected intravenously *via* the lateral tail vein. SPECT/CT images were acquired at 1 h and the next day either at 16 h or 24 h after injection, including

one mouse undergoing dynamic acquisition after injection using 5 min frames. After the final imaging session, mice were euthanised by cervical dislocation and selected organs, tissues and blood were removed and transferred into pre-weighed counting tubes. The weight of and amount of radioactivity in each was measured using a HiDex gamma counter (Lablogic). Counts were converted into radioactivity units (MBq) using a calibration curve generated from known standards. All values were decay-corrected to the time of injection, and the percentage of the injected dose per gram (%ID/g) of each sample was calculated. Five animals were used per group. Flash-frozen xenograft tissue from mice was cyrosectioned (to 10 µm sections) and analysed by autoradiography using a Cyclone Plus Phosphor Imager (PerkinElmer). Adjacent *ex vivo* tissue sections were probed by immunofluorescence for γH2AX foci formation and compared with xenograft tissue obtained from treatment-naive mice.

In a separate study, CA20948 subcutaneous xenografts were established in the right hind flank of female athymic BALB/c *nu/nu* mice as above. Xenograft-bearing mice were administered ^{177}Lu-DOTATATE (20 MBq, 0.33 µg) in sterile PBS (100 µL), or PBS only, by intravenous injection *via* the lateral tail vein. SPECT/CT images were acquired at 1, 24, 48, 72 h post ^{177}Lu-DOTATATE injection. In addition, mice were administered ^{111}In-anti-γH2AX-TAT (5 MBq, 5 µg), or ^{111}In-IgG-TAT (5 MBq, 5 µg) in sterile PBS (100 µL) injected intravenously immediately after the 1 h imaging session *via* the lateral tail vein (see scheme below). After the final imaging session, mice were euthanised by cervical dislocation and selected organs, tissues and blood were removed and transferred into pre-weighed counting tubes with radioactivity spectra measured using a HiDex automated gamma counter. Activities for each isotope were calculated from area under curve analysis using GraphPad prism, based upon standard curves of mixtures containing known activities of lutetium-177 and indium-111. All values were decay-corrected to the time of injection, and the percentage of the injected dose per gram (%ID/g) of each sample was calculated. Five animals were used per group.

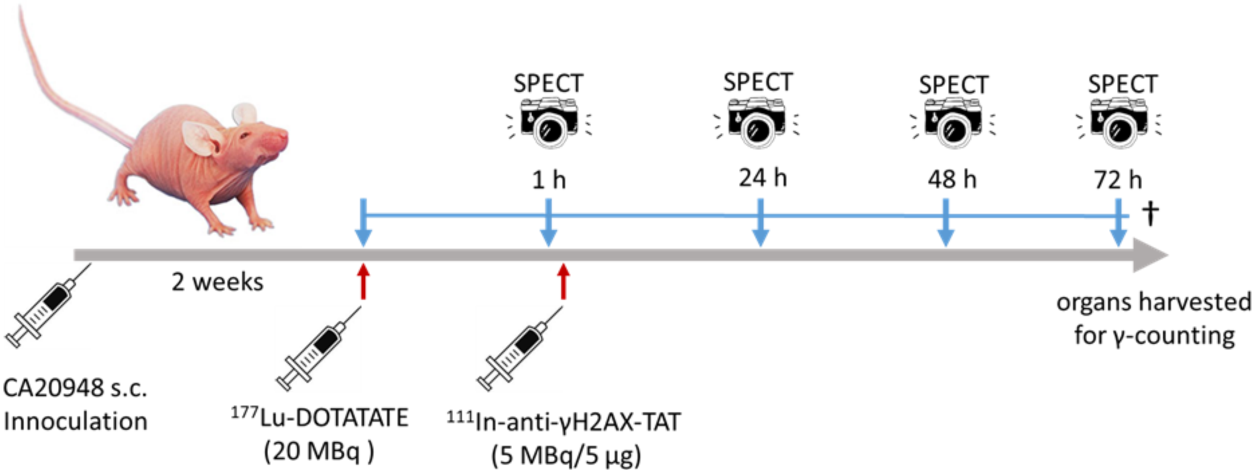

Supplemental Fig. 1: Schematic representation of the therapy and imaging protocol.

In vivo ^{177}Lu dosimetry

The absorbed doses in the tumour xenografts by ^{177}Lu-DOTATATE were determined by using the spheres dosimetry S-values from the IDAC code (*9*). The absorbed dose rate at each time-point was calculated by multiplying the activity at that time-point with the ^{177}Lu S-value interpolated according to their initial volume, assuming a mass density of 1.03 g/cm^3 (lympatic nodes). The tumour time-activity data were fitted with a single exponential curve using Graphpad prism. At each imaging time-point the absorbed dose was calculated by integrating the exponential time-activity curve from time of injection (t=0) to the imaging time-point to obtain the cumulative activities as well as to infinity. Following the MIRD-scheme the absorbed dose is the product of the cumulated activity and the S-value.

Ex vivo immunofluorescence and autoradiography

Sections of tumour xenograft were obtained at 10 µm thickness using a cryostat (CM1950, Leica Biosystems). Tissue sections were allowed to reach room temperature and were then washed in PBS and fixed using 4% paraformaldehyde solution for 10 min. After washing, the sections were permeabilised with 0.2% triton/TBS for 10 min at room temperature, washed again with TBS and blocked in 2% BSA in TBST with glycine 0.3 M and 5% goat serum for 1 h in a flat humid chamber at room temperature. Primary anti-γH2AX antibody (JBW301, Millipore), diluted (1:800) in blocking buffer (minus glycine) was applied directly to the slide and incubated in a humid chamber at 4°C overnight, and then washed with TBST. Fluorescently labelled secondary antibody, 1:250 dilution of a goat anti-rabbit IgG Alexa Fluor® 488 (Invitrogen A11034) was applied directly to the slide and incubated in a humid chamber at room

temperature for 1 h. After further washing, excess fluid was removed and a small drop of Vectashield plus DAPI (Vector H-1200) was applied to each section and a coverslip placed on the slide and secured with Covergrip (Biotium 23005). Fluorescence micrographs were acquired on an Andor Dragonfly microscope (Oxford Instruments) using a 40x/1.3 oil lens. Autoradiography was performed on adjacent sections, using a digital autoradiography platform (Cyclone, Perkin Elmer). Micrographs were registered to autoradiographs using a landmark-based MATLAB algorithm developed in-house. Coincidence of anti-γH2AX antibody associated fluorescence with autoradiography signal was performed on binned co-registered adjacent slides using rigid registration with the JaCOP (Just Another Co-localisation Plugin) in ImageJ.

Supplementary Tables

Supplemental Table 1: Estimated α and β values for linear quadratic equations fitted to clonogenic survival curves in a panel of six cell lines after either exposure to increasing doses of ^{177}Lu-DOTATATE for 2 h, or EBRT. Since clonogenic survival of U2OS, QGP-1, BON-1 and H727 were not significantly affected by ^{177}Lu-DOTATATE (plating efficiency >90%), α and βg values could not be calculated.

cell line	EBRT	^{177}Lu-DOTATATE
**CA20948**	$\alpha = 0.18 \pm 0.01$, $\beta = 0.038 \pm 0.001$	$\alpha = 0.63 \pm 0.07$, $\beta g < 10^{-6}$
**U2OS**	$\alpha = 0.34 \pm 0.04$, $\beta = 0.017 \pm 0.005$	NA
**U2OS^{SSTR2}**	$\alpha = 0.34 \pm 0.02$, $\beta = 0.01 \pm 0.002$	$\alpha = 0.16 \pm 0.03$, $\beta g < 10^{-6}$
**QGP1**	$\alpha = 0.18 \pm 0.01$, $\beta = 0.0063 \pm 0.0007$	NA
**BON1**	$\alpha = 0.32 \pm 0.02$, $\beta = 0.017 \pm 0.003$	NA
**H727**	$\alpha = 0.06 \pm 0.01$, $\beta = 0.027 \pm 0.001$	NA

Supplemental Table 2: Accumulated absorbed radiation dose from ^{177}Lu in tumour tissue, after administration of ^{177}Lu-DOTATATE (20 MBq, 0.33 µg) in CA20948-tumour-bearing mice.

IDAC	Absorbed dose (Gy)									
	^{177}Lu-DOTATATE / ^{111}In-IgG-TAT					^{177}Lu-DOTATATE / ^{111}In-anti-γH2AX-TAT				
**Time (h)**	m1	m2	m3	m4	m5	m6	m7	m8	m9	m10
**1**	0.27	0.36	0.24	0.22	0.33	0.38	0.26	0.28	0.24	0.31
**24**	5.5	7.5	4.6	4.4	6.8	8.1	5.0	5.7	4.7	6.5
**48**	9.5	13.0	7.7	7.3	11.6	14.2	8.0	9.8	7.5	11.2
**72**	12.3	17.0	9.7	9.3	15.1	18.8	9.8	12.8	9.3	14.6
**infinity**	19.4	28.1	13.4	13.1	24.2	32.8	12.5	20.4	12.0	23.9

Supplemental Figures

Supplemental Fig. 2: ^{111}In/^{177}Lu dual SPECT imaging using the MILabs VECtor[4] small animal SPECT/CT scanner, of a series of 1.5 mL tubes filled with 50-250 kBq or ^{177}Lu and 50 kBq of ^{111}In.

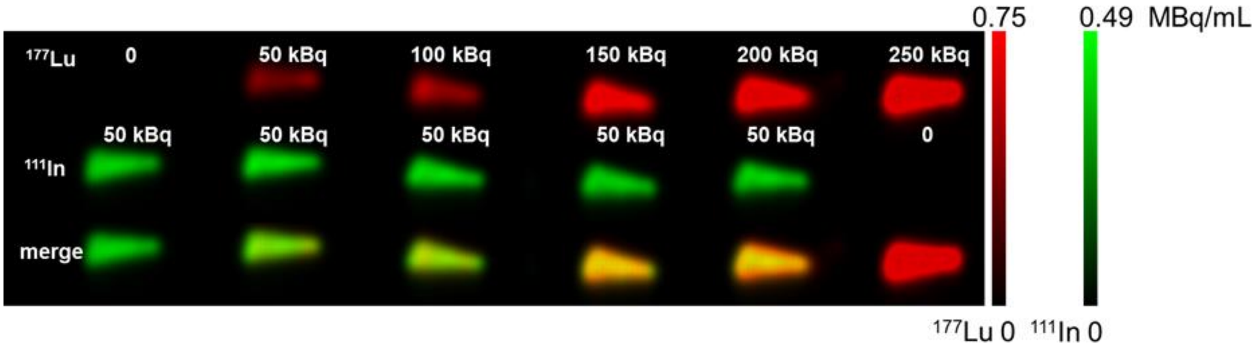

Supplemental Fig. 3: Clonogenic survival following *in vitro* exposure of cancer cell lines to varying amounts of ^{177}Lu-DOTATATE, or increasing amounts of external beam radiation therapy. **(a)** QGP1 cells, **(b)** BON1 cells, or **(c)** H727 cells. Radiation absorbed doses for ^{177}Lu were determined based on ^{177}Lu uptake data obtained from a separate study (Supplementary Figure S3).

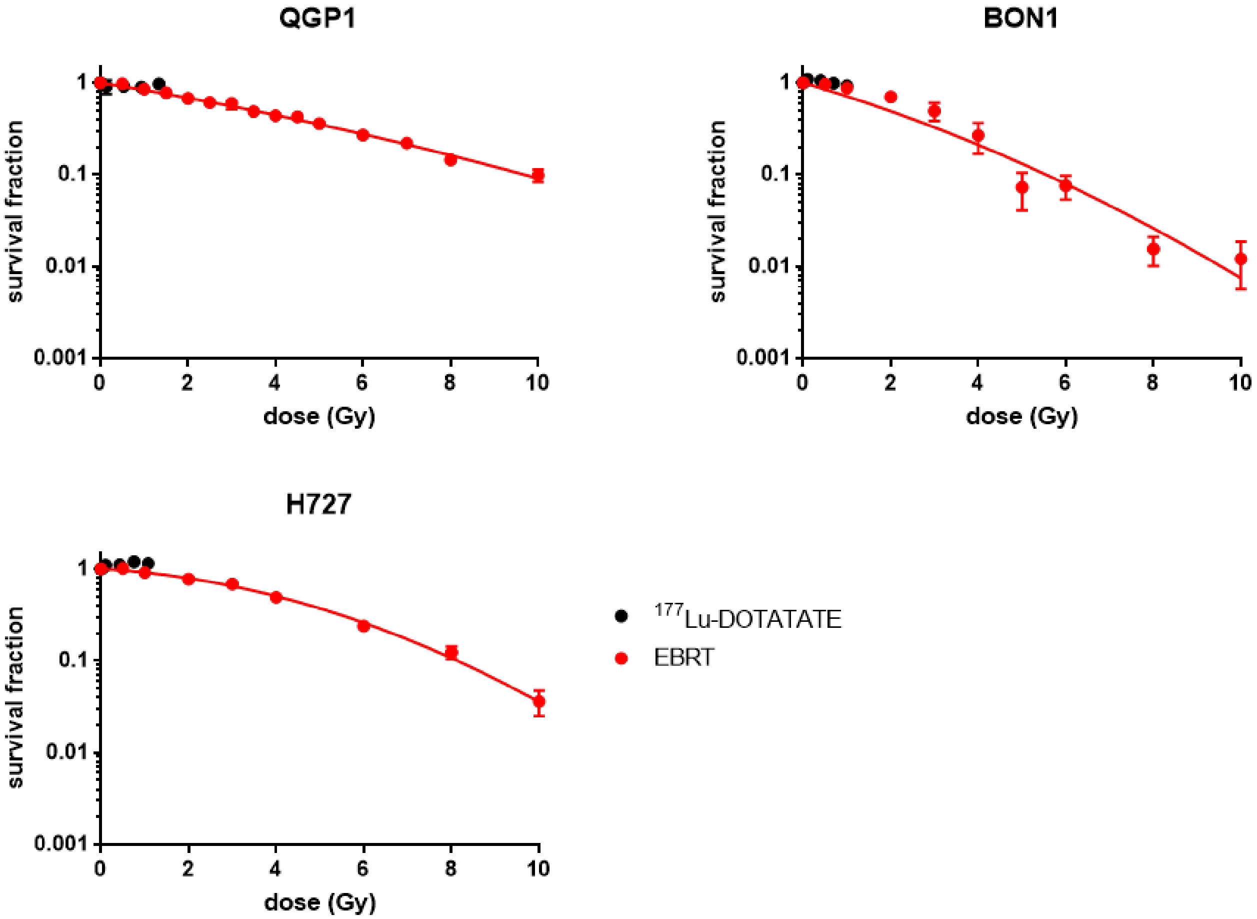

Supplemental Fig. 4: *In vitro* uptake of ^{177}Lu-DOTATATE in a panel of cell lines. Aliquots of 2×10^5 cells were exposed to ^{177}Lu-DOTATATE (2.5 MBq/mL, 50 MBq/nmol, in 200 μL) for up to 24 h at 37°C. The amount of ^{177}Lu associated with the cell membrane, cytoplasm and cell nuclei was determined after cell fractionation. Please note the difference in Y-axis scales.

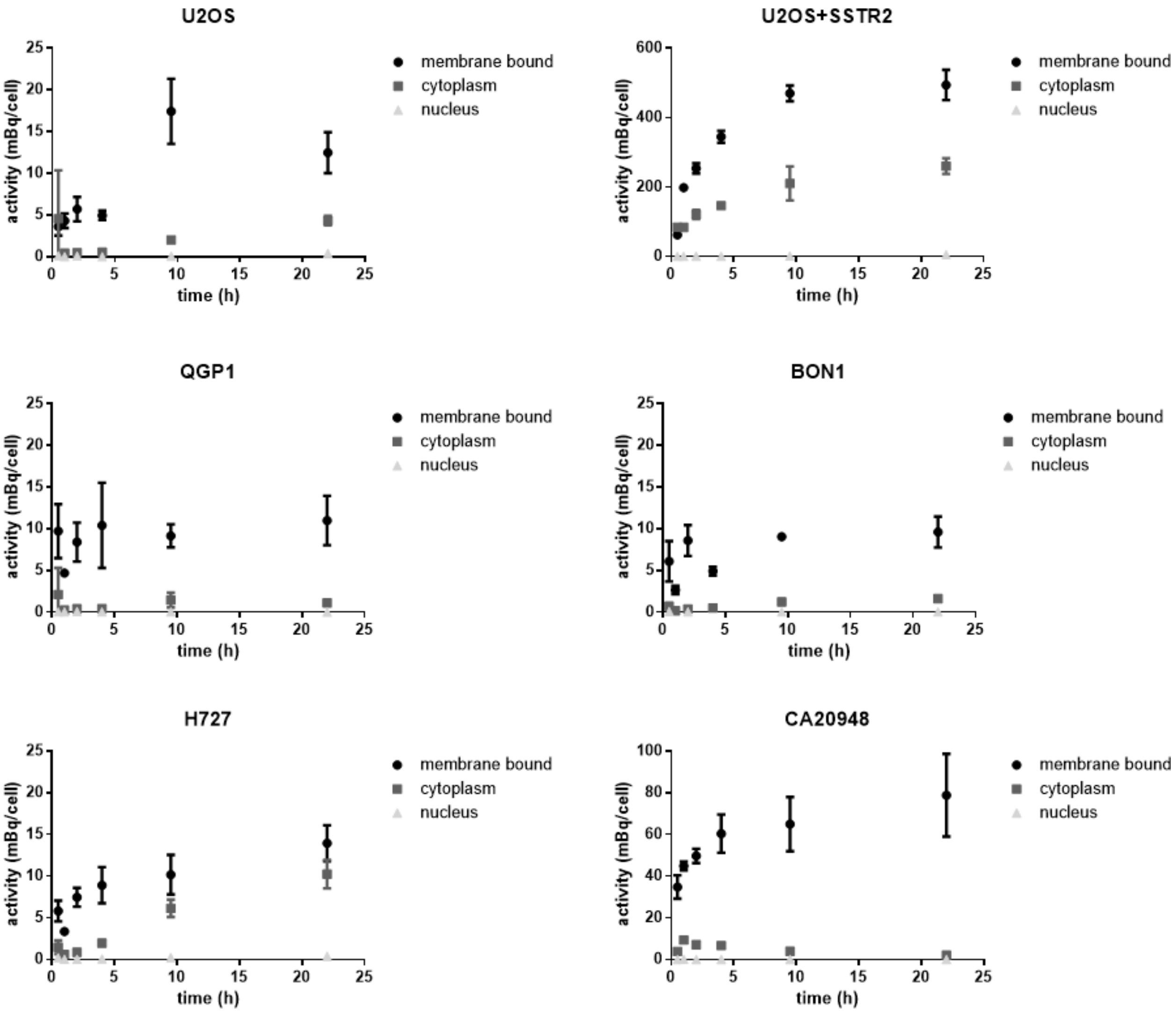

Supplemental Fig. 5: The number of γH2AX foci per cell was determined at various intervals after exposure of U2OS^{sstr2} or wild type U2OS cells to ^{177}Lu-DOTATATE for 2 h, or after EBRT (6 Gy).

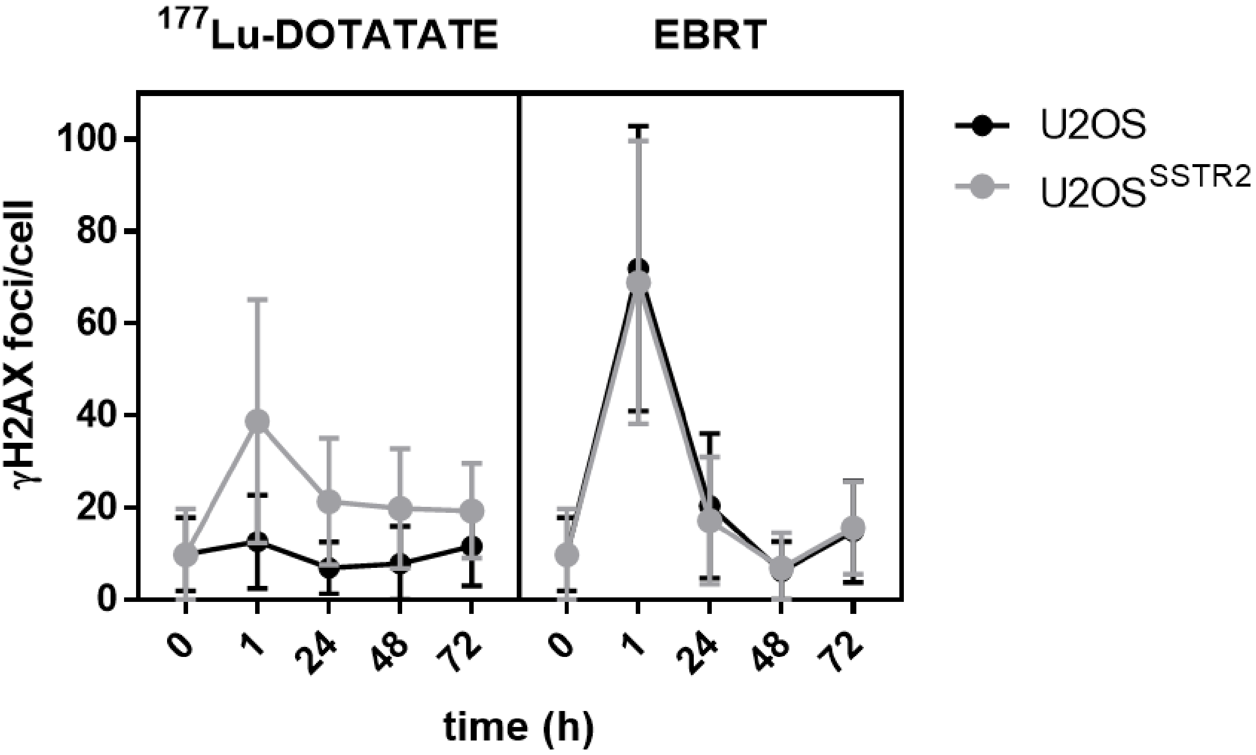

Supplemental Fig. 6: (a) Distribution of ^{177}Lu in tumour, blood and selected tissues, 24 h after intravenous administration of ^{177}Lu-DOTATATE (20 MBq, 0.33 µg) to mice bearing QGP1, H727, BON1, or CA20948 xenograft tumours (n = 5). **(b)** Representative VOI analysis of dynamic SPECT imaging of ^{177}Lu uptake in a CA20948 tumour xenograft within the first 110 min after administration.

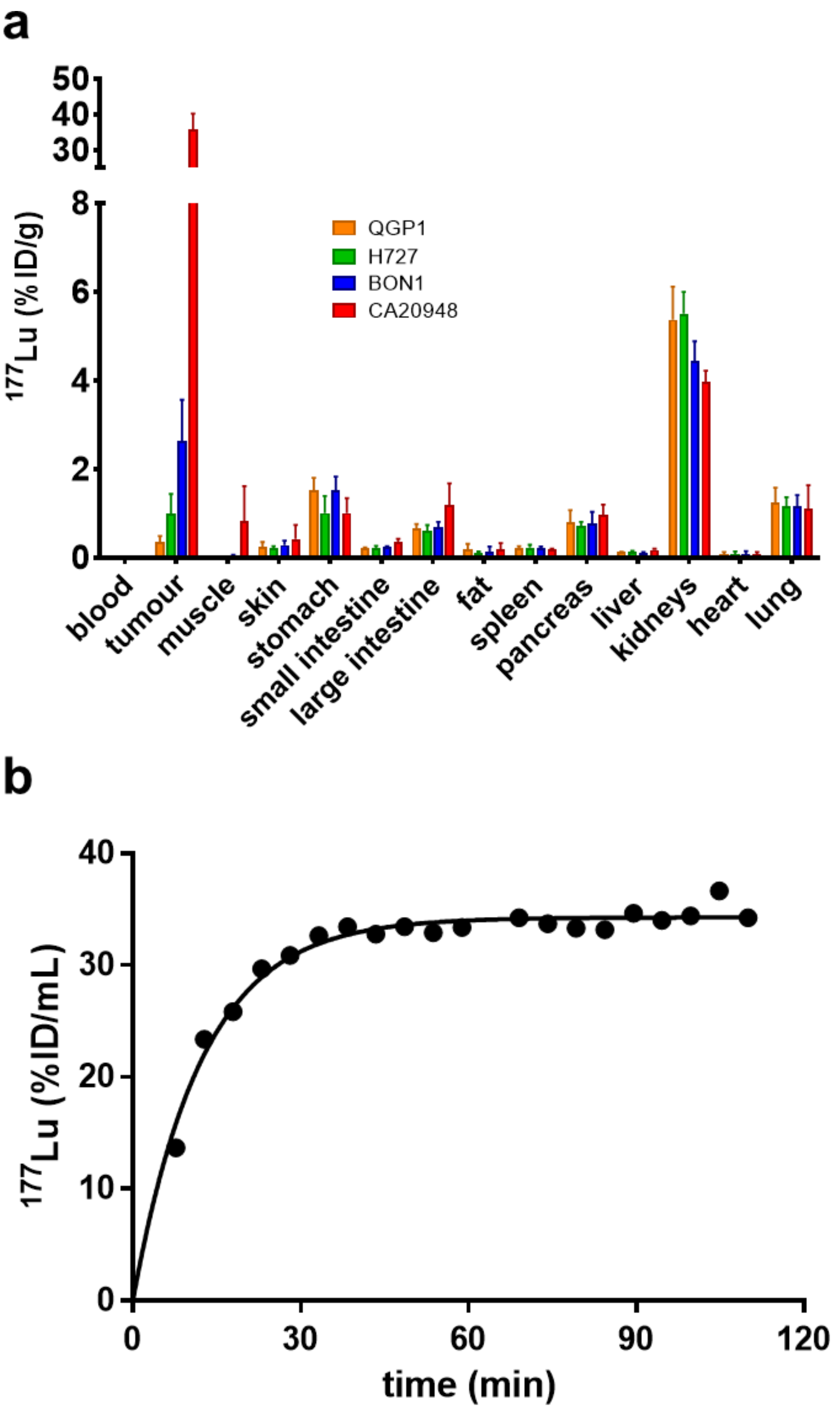

Supplemental Fig. 7: Representative Autoradiography of ^{177}Lu and γH2AX immunohistochemistry of tumour xenograft sections harvested, 72 h after intravenous administration of ^{177}Lu-DOTATATE (20 MBq, 0.33 µg) in a CA20948 xenograft-bearing athymic mouse. As in Fig. 3A and 3C. Three additional examples of ^{177}Lu-DOTATATE uptake in tumour sections, correlated with γH2AX expression. (scale bar = 500 µm)

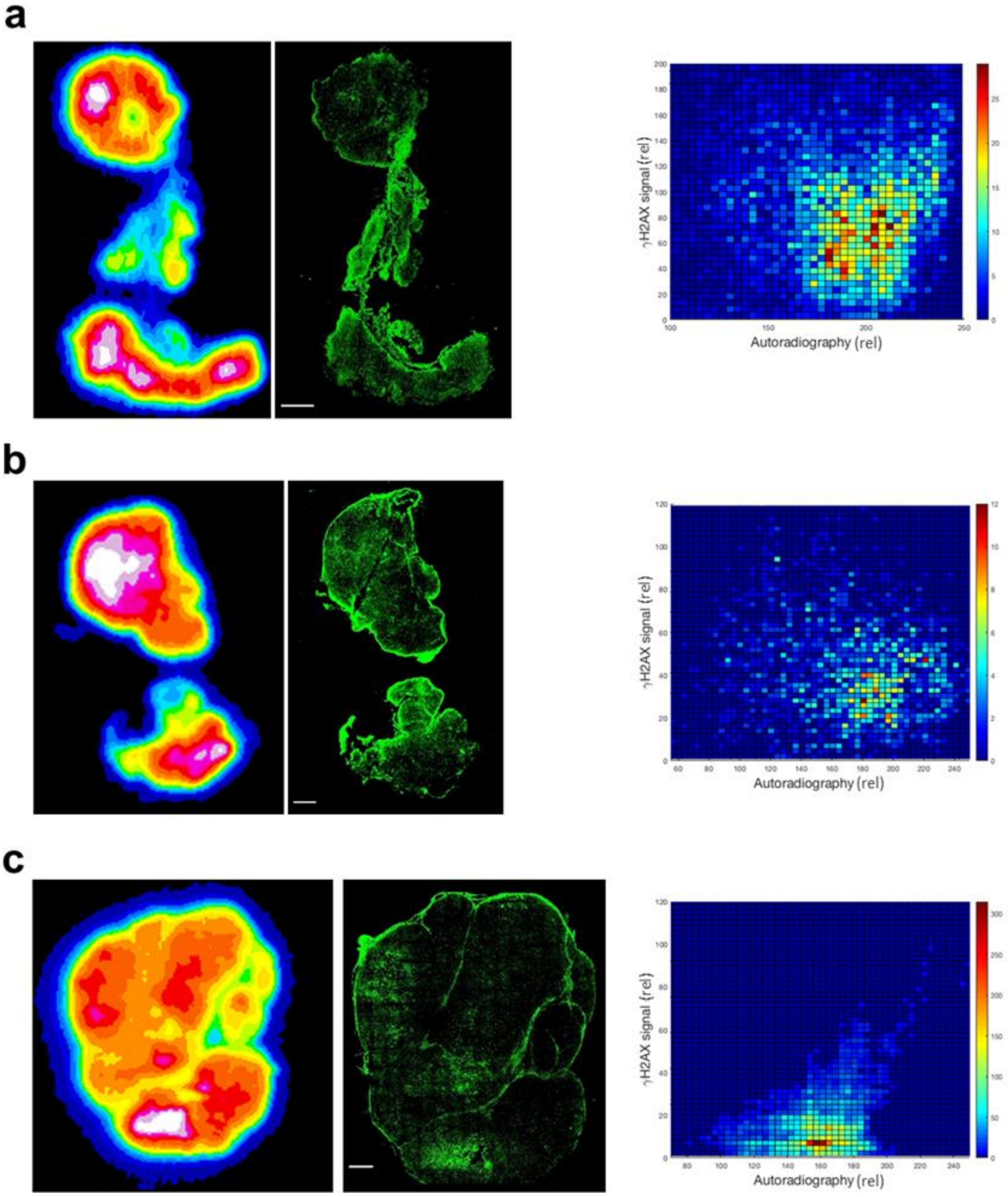

Supplemental Fig. 8: As in Fig. 4A. Tumour uptake in individual mice bearing CA20948 xenografts of ^{111}In-anti-γH2AX-TAT or ^{111}In-IgG-TAT at various times after treatment of mice with ^{177}Lu-DOTATATE (20 MBq, 0.33 μg) **(a)** or vehicle control **(b)**.

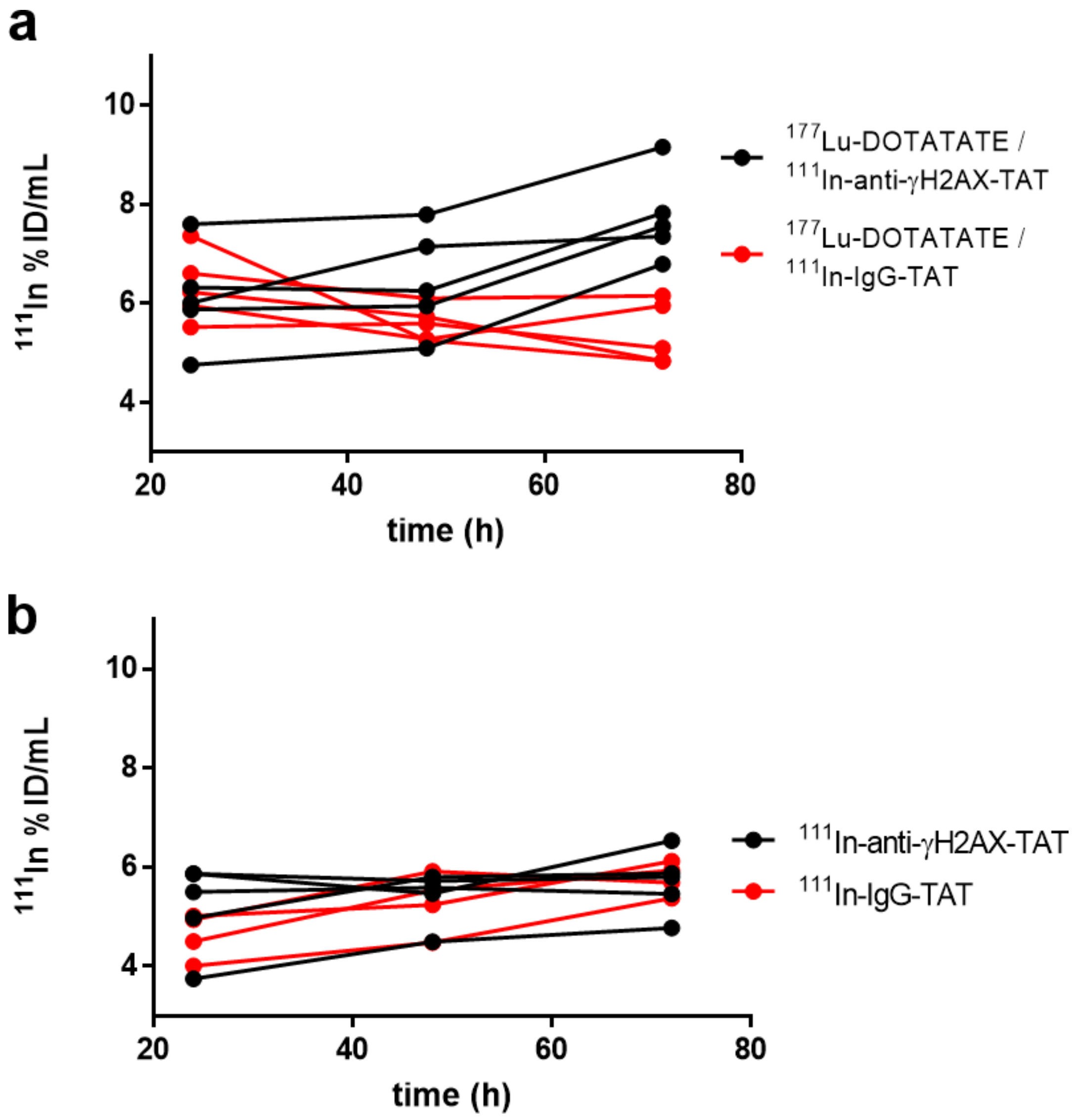

Supplemental Fig. 9: (a) Distribution of ^{111}In in blood and selected tissues, 72 h after intravenous administration of ^{111}In-anti-γH2AX-TAT or ^{111}In-IgG-TAT in mice bearing CA20948 xenografts treated with ^{177}Lu-DOTATATE (20 MBq, 0.33 µg) or vehicle control. **(b)** Distribution of ^{177}Lu in ^{177}Lu-DOTATATE-treated animals.

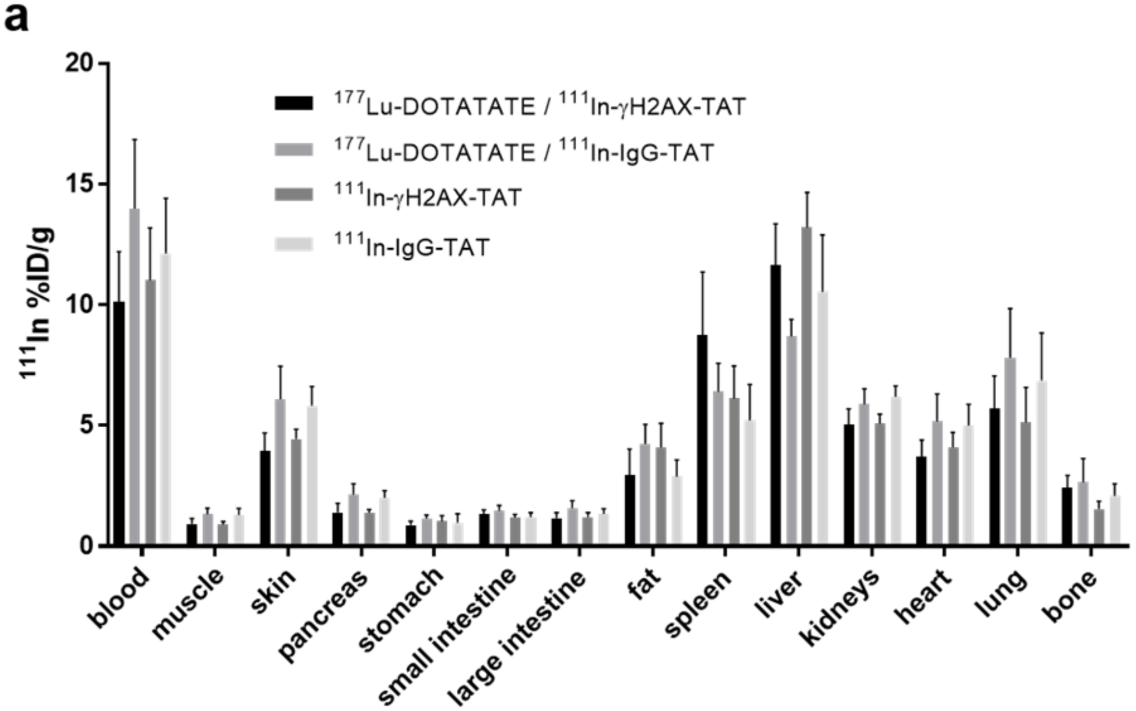

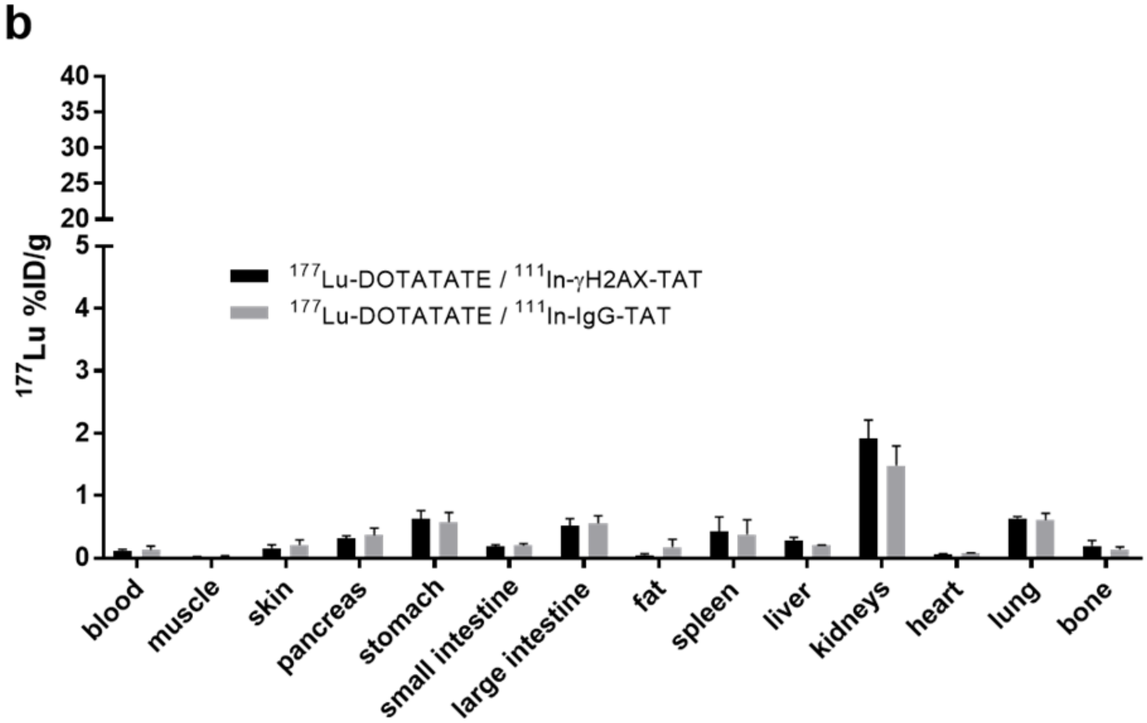

Supplemental Fig. 10: Tumour uptake of ^{177}Lu-DOTATATE time-activity curves, with single exponential curve fits (Pearson $R^2 > 0.94$), the dashed lines indicate the 95% confidence intervals, in mice administered ^{177}Lu-DOTATATE and ^{111}In-IgG-TAT (m1-m5) or ^{177}Lu-DOTATATE and ^{111}In-anti-yH2AX-TAT. S-values for ^{177}Lu were obtained from the spheres model in the IDAC code (lymphatic nodes).

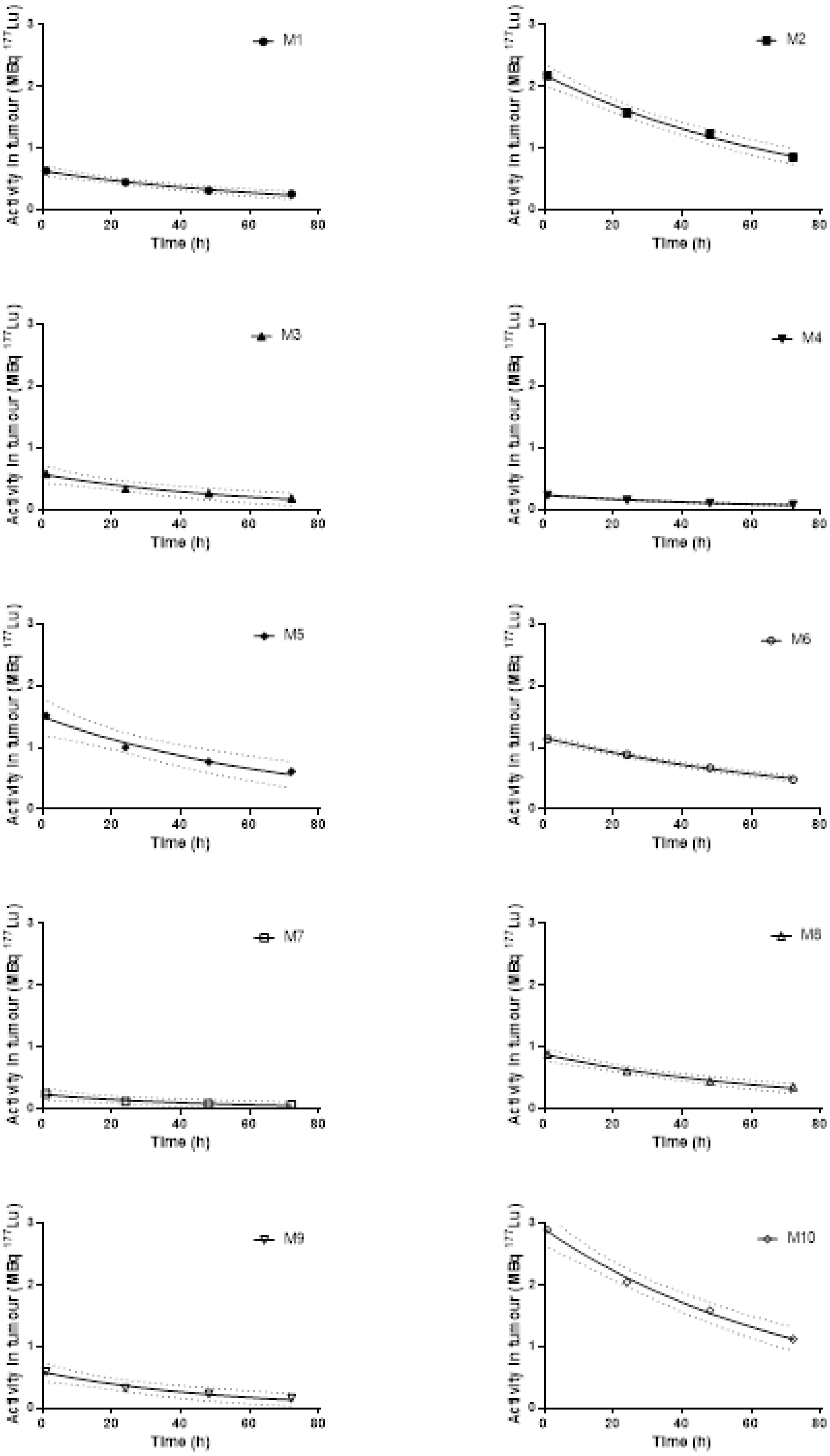

References for Supplementary Material

1. Dalm SU, Nonnekens J, Doeswijk GN, et al. Comparison of the Therapeutic Response to Treatment with a 177Lu-Labeled Somatostatin Receptor Agonist and Antagonist in Preclinical Models. *J Nucl Med.* 2016;57:260-265.

2. Bernard BF, Krenning E, Breeman WA, et al. Use of the rat pancreatic CA20948 cell line for the comparison of radiolabelled peptides for receptor-targeted scintigraphy and radionuclide therapy. *Nucl Med Commun.* 2000;21:1079-1085.

3. Cornelissen B, Hu M, McLarty K, Costantini D, Reilly RM. Cellular penetration and nuclear importation properties of 111In-labeled and 123I-labeled HIV-1 tat peptide immunoconjugates in BT-474 human breast cancer cells. *Nucl Med Biol.* 2007;34:37-46.

4. Goddu SM, Howell RW, Rao DV. Cellular dosimetry: absorbed fractions for monoenergetic electron and alpha particle sources and S-values for radionuclides uniformly distributed in different cell compartments. *J Nucl Med.* 1994;35:303-316.

5. Salvat F, Fernandez-Varea JM, Sempau J. *PENELOPE-2011: A Code System for Monte Carlo Simulation of Electron and Photon Transport*: OECD Nuclear Energy Agency, Issy-les-Moulineaux; 2011.

6. Lee BQ, Nikjoo H, Ekman J, Jonsson P, Stuchbery AE, Kibedi T. A stochastic cascade model for Auger-electron emitting radionuclides. *Int J Radiat Biol.* 2016;92:641-653.

7. Falzone N, Fernandez-Varea JM, Flux G, Vallis KA. Monte Carlo Evaluation of Auger Electron-Emitting Theranostic Radionuclides. *J Nucl Med.* 2015;56:1441-1446.

8. Falzone N, Lee BQ, Able S, et al. Targeting Micrometastases: The Effect of Heterogeneous Radionuclide Distribution on Tumor Control Probability. *J Nucl Med.* 2018.

9. Andersson M, Johansson L, Eckerman K, Mattsson S. IDAC-Dose 2.1, an internal dosimetry program for diagnostic nuclear medicine based on the ICRP adult reference voxel phantoms. *EJNMMI Res.* 2017;7:88.
